# Coronary angiogenic effect of long-term administration of *Nigella sativa*

**DOI:** 10.1186/s12906-017-1795-z

**Published:** 2017-06-13

**Authors:** Lubna I. Al Asoom

**Affiliations:** College of Medicine, University of Dammam, 31441, Dammam, 1982 Saudi Arabia

**Keywords:** Coronary angiogenesis, *Nigella sativa*, Exercise training, VEGF, VWF, PECAM-1

## Abstract

**Background:**

Coronary angiogenesis is one of the preferable adaptive responses of aerobic training. Previous studies found inotropic and hypertrophic cardiac effects for long-term administration of *Nigella sativa* (*NS*), but no studies have explored its coronary angiogenic effect. The present study compared the effect of long-term *NS*- administration and exercise training on the induction of coronary angiogenesis.

**Method:**

Fifteen adult male Wistar rats were divided into three groups: control, *NS*-fed, and exercise-trained (Ex). The *NS*-fed rats were administered 800 mg/Kg *NS* orally for eight weeks. The (Ex) rats were trained on a five-lane treadmill at a speed of 18 m/min and a grade of 32° for two hour/day for eight weeks. After the experiment, the hearts were extracted and immunohistological slides were prepared using rat vascular endothelial growth factor (VEGF), platelet endothelial cell adhesion molecule-1 (PECAM-1), Von Willebrand factor (VWF) and nitric oxide synthase-2 (NOS-2) antibodies (Ab). Photomicrographs were analysed using ImageJ software, and the % of the immunostained-area of 10 fields per specimen was recorded.

**Result:**

VEGF was significantly higher in the *NS*- (2.59±1.37%) and Ex rats (2.51±1.86%) compared to the control group (1.58±0.78%) with *P*<0.01. The VWF was significantly lower in the two experimental groups (1.57±0.83%, 1.07±0.72%) for *NS* and Ex groups respectively, compared to the controls (2.38±1.72) with *p*<0.01. Only Ex group had a higher PECAM-1 (1.79±0.78%) and lower NOS-2 (0.83±0.57%) than the control group (1.19±1.17%, 1.25±1.19%) for PECAM-1 and NOS-2 with *P*<0.01 and *P*<0.05 respectively.

**Conclusions:**

The present study demonstrated an increase in VEGF and a decrease of the VWF in the hearts of *Nigella*-fed and exercise-trained rats. This might indicate the potentiality for induction of coronary angiogenesis via long-term administration of *NS* and exercise training. *NS* effect on coronary angiogenesis needs to be explored further as it might lead to a new promising preventive and therapeutic agent of the ischemic heart disease.

## Background

Coronary artery disease (CAD) is one of the leading causes of death worldwide. The World Health Organization (WHO) reports that 31% of deaths across the globe are due to cardiovascular diseases, and ischemic heart disease accounts for 46% of these deaths [1]. The current prevalence and consequences of CAD might also increase dramatically in the future due to the uncontrolled increment of multiple risk factors, including obesity, hypercholestrolemia, hypertension, diabetes and smoking [2].

Exercise training has long been recommended as a cardiovascular protective measure. Favourable cardiac and coronary adaptations to exercise training are well known, and they have been reported in the literature [3, 4]. Exercise training can culminate in the enhancement of myocardial blood flow through multiple structural and physiological alterations of the coronary vascular bed. This includes increments in the conduit of large arteries, increases in the number of arterioles and induction of capillary growth [5]. A wide range of stimuli have been suggested as explanations for the angiogenic effect of exercise training. During acute bouts of exercise, the increased myocardial blood flow leads to increased shear stress and the release of endothelial vasodilators and nitric oxide [6]. Growth factors such as fibroblast growth factors (FGFs), vascular endothelial growth factor (VEGF) and insulin like growth factor-I (IGF-I) have been found to be upregulated in the coronary circulation in exercise-trained animals [7].

Assessment of increases in the coronary circulation in exercise-trained human subjects and animals varies. In exercise-trained human subjects and athletes, coronary circulation capacity is assessed by evaluating coronary flow, using electromagnetic flowmeter or ultrasonic devices [8]. In animals, immunohistochemical studies are used to label the vascularity and measure the cross-sectional area [9].

Despite accumulating evidence of the prophylactic influence of exercise training on cardiac health and coronary circulation, the compliance toward training is low worldwide. Adjuvant therapeutics aimed to induce angiogenesis are of compelling need either as prophylaxis of ischemia or as an attempt to rescue ischemic tissue.

The black seed is a herbaceous plant used as a traditional remedy in the Arab countries, Iran and India. It received its name from the appearance of the seeds which are small (1–5 mg in weight) and have dark grey to black colour [10]. Generically, it is known as *Nigella sativa Linn* (NS). The main constituents of *NS* are crude fat (30–40%), crude protein (20–30%), crude fibre (5–10%) and multiple minerals including potassium, Magnesium, calcium, phosphorus and sodium. The crude fat is composed of fixed and volatile oil and it contains wide range of fatty acids such as linoleic, linolenic, oleic and palmitic acids. Thymoquinone was isolated and recognized as an important and active constituent of both the volatile and fixed oil [11].

*NS*and its active component (thymoquinone) demonstrated multiple therapeutic effects in multiple studies. The therapeutic effect included anti-inflammatory [12], antioxidant [13], antibacterial [10], hypoglycemic and antilipidemic effects [14, 15]. Furthermore, available evidences showed multiple favourable cardiovascular responses of *NS*. Two previous studies reported antihypertensive effect of *NS* on humans and animals [16, 17]. Positive cardiac adaptation for long term administration of *NS* was also demonstrated. In a study performed by El Bahai et al. [18], long-term administration of *NS* for two months to adult Wistar rats, led to an increase in the peak force of cardiac contraction, myocardial flow rate and the rate of tension development [18]. Another study performed by the same group, showed structural remodelling of the myocardium, including myocardial hypertrophy manifested by increased heart weight/body weight (HW/BW), left ventricular weight/body weight (LVW/BW) and increased diameters of cardiomyocytes [19]. The histological and electrophysiological characteristics of hypertrophied hearts induced by *NS* were compared to exercise-induced cardiac hypertrophy, and multiple similarities were found [19, 20]. Furthermore, Ansam et al. reported a protective effect of *NS* against ischemia reperfusion insult in rat hearts when the animals were treated for three months with oral *NS* [21].

The previous reported studies of the cardiac adaptation to long term administration of *NS* drew the attention to a possible preferable coronary circulation response by the same dose of *NS*. Therefore, this present study aims to examine the effect of long-term administration of *NS* on the structural remodelling of coronary circulation, explore some of its underlying mechanisms and compare it to exercise-training induced coronary vascular remodelling.

## Methods

### Animals

Fifteen normal adult male Wistar rats (age: two months, weight: 200–300 g) were obtained from the animal house at the University of Dammam, in Dammam, Saudi Arabia. All the animals were housed individually in labelled cages with adequate ventilation and illumination, and in normal light cycle (12 light/12 dark). Normal laboratory chow and tap water were available ad libitum. The sample size was selected based on similar published work with the consideration of the minimum requirement [22].

Ethical approval was provided by the ethical committee of the Deanship of Scientific Research at Dammam University with reference number IRB-2014-01-165. This committee is a branch of the National Committee of Bioethics, Saudi Arabia. The rats were divided equally and randomly into three groups: the control group, the *NS-*treated group, and the exercise-trained (Ex) group. The *NS*-treated rats were given 800 mg/Kg *NS* daily for eight weeks. *NS* whole seeds were obtained from a local herb store in Dammam, Saudi Arabia. It is a product of the central region of Saudi Arabia. These seeds were previously analysed for its constituents by Al-Jassir [23]. The dose, duration and preparation of *NS* was similar to that used by El Bahai et al. and showed cardiac hypertrophy and positive inotropic effect [18]. Oral administration was done via a feeding needle. A suspension of *NS* was prepared by dissolving 10 g of freshly ground *NS* seeds in 100 ml of distilled water. The animals in the control group were given an equivalent volume of water.

The rats in Ex group were trained on a treadmill (IITC Life Science, five-lane rat treadmill), five days/week for eight weeks. The speed, grade and duration were progressively increased during the first week until the final protocol was achieved, with a speed of 18 m/min and an inclination of 32°, for a two-hour session [24]. An electric grid at the rear of the belt was used as the running stimulus. An equivalent volume of water was also administered to this group.

### Extraction of the hearts

The rats were anaesthetized with a ketamine cocktail (60% ketamine, 40% xylasine). A dose of 0.2 ml/250 g body weight was injected intraperitoneally. Hearts were extracted through longitudinal abdominal incisions and soaked in cold Ringer’s solution. Excess connective tissue and great vessels were removed. Lumens were rinsed with Ringer’s solution to remove excess blood and clots. Finally, the hearts were blotted dry and weighed. The right ventricles and both atria were removed, and the remaining left ventricles were weighed. The free wall of the left ventricles was excised and stored in 4% formal-saline for histological preparation.

### Preparation of the light microscope slides

The left ventricular free wall was initially fixed in 4% formal-saline. Later, the specimens were washed briefly in water, and then labelled and placed in a tissue processor (Tissue-Tek VIP) overnight. The specimens were dehydrated using ascending grades of alcohol: 70%, 90% and 100% two changes, and two changes of xylene for a period of two hours, respectively.

The process of embedding was initiated. The specimens were impregnated in two changes of molten paraffin wax for a period of two hours for each change, and they were subsequently embedded in molten paraffin wax at a temperature of 60 °C. Cassettes were used to control the position of the specimens. The blocked tissues were labelled and allowed to solidify; they were then sectioned using microtomy (LEICA RM 2235, Leica BioSystems Buffalo Grove, IL, USA) at a thickness of 3 um. The sectioned tissues were floated in warm water, and then placed on microscope slides, labelled and allowed to dry.

The sections were dewaxed, washed in water and stained using the following antibodies:

- Rat VEGF 164 affinity purified polyclonal antibody (Ab), Goat IgG (R&D Systems, Minneapolis, MN, USA) [25].
- Anti CD31 (PECAM-1) for endothelial cells to determine the density of the capillaries, purified polyclonal Ab, Goat IgG (R&D Systems, Minneapolis, MN, USA) [26].
- Anti Von Willebrand factor (VWF) antibody purified polyclonal Ab, Goat IgG (R&D Systems, Minneapolis, MN, USA) [9].
- Anti NOS-2 antibody staining, using a commercially available kit (Novostain Super) ABC Kit (universal), NCL-ABCu (Novocastra Laboratories, Ltd., Newcastle upon Tyne, UK).

### Estimation of the antibody labelling in the light microscopic slides

Each rat specimen was stained using all the antibodies listed above. Estimation of the immunostained area of each antibody was performed using photomicrographs obtained via a digital microscope (Coolscope, Nikon Instruments Europe BV, Amsterdam Netherlands). Ten fields were selected randomly from each section. All the selected fields were clear from freezing defects, gaps or folds. The overlap of fields was clearly avoided. The slide selection and analysis was conducted by a blind operator. The sections were labelled with numbers that were unknown to the operator.

**Fig. 1:**
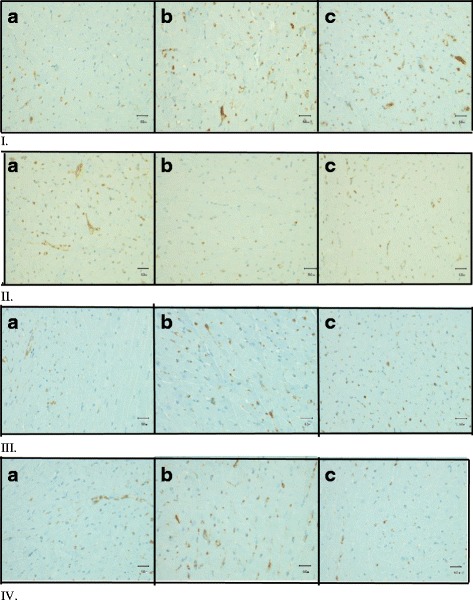
Photomicrographs illustrated immunohistochemistry study of left ventricular tissue labeled by **I**. VEGF, **II**. Von Willebrand **III**. PECAM-1, **IV**. NOS-II Antibodies obtained from: **a**. control, ***b*** *. nigella sativa* fed rats, **c**. exercise trained rats

Image J software was used to analyse the sections. The total immunostained area in each field was measured in pixels and expressed as percentage of the total field area (%). The mean value of the immunostained area for all fields of a specific antibody in each group was obtained [22]. (see Fig. 1 for the photomicrographs of all antibodies).

### Statistical analysis

IBM - Statistical analysis software package - SPSS version 20 was used to analyse the data. All data were expressed as mean ± SD. Analysis of variance (ANOVA) and LSD post-hoc test were used to compare data among the groups. The level of significance was set at *p* < 0.05.

## Results

All rat groups were matched in regard the initial and the final body weight.

There was no significant difference in HW, LVW, and LVW/HW between the groups. No difference were also found between the heart weight and left ventricular weight when normalized to body weight and tibial length. (Data are not shown).

**Fig. 2:**
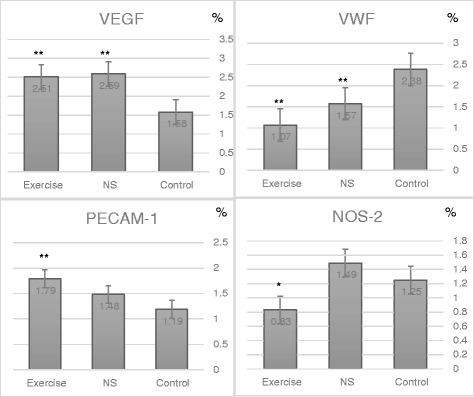
Comparison of the angiogenic growth factors and antigens (VEGF, VWF, PECAM-1, and NOS-2) between the experimental groups: Control, *NS* (*Nigella sativa* fed), Exercise (Exercise trained), *P < 0.05, **P < 0.01

VEGF was significantly higher in the Nigella-fed (2.59 ± 1.37%) and the exercise-trained (2.51 ± 1.86%) groups compared to the control (1.58 ± 0.78%), *P* < 0.01. VWF was significantly lower in the Nigella-fed (1.57 ± 0.83%) and the exercise-trained (1.07 ± 0.72%) groups compared to the control (2.38 ± 1.72%), *P* < 0.01. Only the exercise-trained group was found to have a higher PECAM-1 (1.79 ± 0.78%) and a lower NOS-2 (0.83 ± 0.57%) than the control (PECAM-1: 1.19 ± 1.17%, NOS-2:1.25 ± 1.19%), *P* < 0.01, *P* < 0.05 respectively (Fig. 2).

## Discussion

The present study aimed to explore the effect of long-term administration of *NS* on coronary circulation in Wister rats using immunohistochemical methods. The immunohistological slides were prepared using PECAM-1, to target endothelial cell antigens, VEGF, known to induce capillary growth, VWF that regulates and limits vascular growth, and NOS-2 which is responsible for the production of nitric oxide (NO). Analysis of the data revealed a significant increase in VEGF in both the *NS*-fed and the Ex-trained groups, and a significant decrease in the VWF in the *NS*-fed and the Ex-trained groups. In addition, only the exercise-trained group showed significant increase in PECAM-1 and a significant decrease in NOS-2.

VEGF is a growth factor that induces the sprouting of the capillaries. It does so by dissolving the basement membrane of the existing capillaries, and then stimulating the proliferation and migration of endothelial cells [27]. VEGF interacts with the endothelial cell adhesion molecules during the process of endothelial growth and proliferation [28]. In a previous study reported by Marini et al., exercise-trained rats for four weeks showed upregulation of VEGF, accompanied simultaneously with capillary sprouting and capillary angiogenesis [9]. Induction of VEGF production in an endothelial cell culture has been found to promote cell division and cell proliferation [29]. Administration of VEGF genes into porcine myocardium induced angiogenesis and increase the microvascular bed about 1.8 folds as demonstrated by Nurro et al. [30].

In the present study, VEGF was significantly high and comparable in the *NS*-fed and the Ex-trained groups, indicating that both *NS* administration and exercise training are potential factors for the induction of capillary growth.

The process of angiogenesis is also governed by antiangiogenic factors. VWF is a glycoprotein that is synthesised in the endothelial cells, and it is known for its essential haemostatic effect. It stimulates the adhesion and aggregation of platelets in injured tissue, and it acts as a carrier of coagulation factor VIII [31]. VWF plays a role in the regulation of angiogenesis, limits the release of angiogenic factors, such as VEGF, and stabilises the capillary bed [32]. In VWF deficiency, abnormal growth of vessels has been found in a condition known as angiodysplasia [33]. Inhibition of the release and synthesis of VWF by interfering with its genetic expression in mice leads to enhanced angiogenesis, increased VEGF receptor-2 dependent proliferation and migration of endothelial cells [32]. In this study, all the experimental groups (the *NS*, and Ex groups) demonstrated a significantly lower VWF expression level than the control. This finding might support the angiogenic effect of both long-term administration of *NS* and exercise training on coronary circulation. It also supports the similarities in the actions of these two interventions.

NO is a multifunctional cellular signalling molecule, known for its vasodilation action. Recently, evidence has shown that NO plays a role as a mediator of angiogenesis. NO is released from the endothelial cells under the influence of VEGF. VEGF upregulates and activates NOS in endothelial cells [34]. Increased expression of NOS was demonstrated in the hind-limbs of mice after three weeks of wheel running [35]**.** The effect of exercise training on the different isoforms of NOS i.e. endothelial NOS (eNOS), and inducible NOS (NOS-2), might differ. [34]. In a recent study, exercise training stimulated eNOS and reduced iNOS in control rats and normalized iNOS and showed no effect on eNOS in high fat fructose diet rats after ischemia reperfusion injury [36]. In the current experiment, exercise training demonstrated a significant reduction in the expression of NOS-2 in the cross-section of the left ventricles of the exercise-trained rats. A reduction of iNOS might be an indication of cardioprotective effect of exercise training through the mitigation of oxidative stress [37]. eNOS was not assessed in this study due to technical difficulties in providing antibodies with rat species reactivity. *NS* administration showed no effect on NOS-2. Although, *NS* is known to be an anti-inflammatory and antioxidant mediator [15], but it did not show similar effect to exercise training on NOS-2. Other isoforms of NOS might need to be studied under the effect of these two intervention i.e. exercise training and long term administration of *NS* for better comparison of their effects on NOS and NO production.

PECAM-1 is the platelet-endothelial adhesion molecule known as CD31 [38]. The interaction of PECAM-1 with growth factors is essential for angiogenesis. Some studies have used PECAM-1 to estimate capillary density [39, 40]. In a recent study, diabetic rats were exposed to testosterone treatment and exercise training to investigate the effect on these two interventions on neoangiogenesis [30]. That study demonstrated higher PECAM-1 antibody staining after both treatment modalities were administered [41]. Similarly, our study found a significantly higher PECAM-1 density in the Ex-trained group. Our *NS*-fed groups failed to demonstrate a significant increase in PECAM-1. However, the possibility of the presence of ongoing capillary angiogenesis is still valid, as supported by the significant increase in VEGF and the decrease in VWF. A longer duration of *NS* administration might be required to achieve a higher capillary bed density. This finding might be related to the delayed onset of cardiac hypertrophy induced by *NS* due to the lack of continuity of administration of the *NS* treatment during the weekends. Different stages of cardiac and coronary adaptation to exercise training have also been reported [42].

## Conclusion

In conclusion, the present study demonstrated evidence of active coronary angiogenesis induced by eight weeks’ administration of *NS* and aerobic exercise training in adult Wistar rats. This evidence includes a significant increase in VEGF and a reduction of VWF. This finding might be promising for the emergence of a new therapeutic and preventive strategy for CAD in specific and other peripheral artery diseases in general. Further studies are required to explore the potential effect of *NS* on coronary angiogenesis and confirm the present findings.

### Limitations

The present study might be limited by the number of growth factors and antigens tested. Other growth factors known to be involved in the process of angiogenesis whether stimulatory or inhibitory need to be investigated. In addition, all isoforms of NOS need to be assessed and compared. This limitation arises due to the difficulty in recruiting antibodies with good reactivity to rat species.

## Abbreviations

Ab: Antibodies
ANOVA: Analysis of variance
BW: Body weight
CAD: Coronary artery disease
HW: Heart weight
IGF-I: Insulin like growth factor-I
iNOS: Inducible nitric oxide synthase
LVW: Left ventricular weight
NO: Nitric oxide
NOS-2: Nitric oxide synthase-2
NS: *Nigella sativa*
PECAM-1: Platelet endothelial cell adhesion molecule-1
SD: Standard deviation
SPSS: Statistical analysis software package
VEGF: Vascular endothelial growth factor
VWF: Von Willebrand factor
WHO: World health organization

## References

[1] Mendis S, Puska P, Bo N. Cardiovascular disease (CVDs) due to atherosclerosis. In: Global atlas on cardiovascular disease prevention and control: Policies, strategies and interventions. 2011. http://www.world-heart-federation.org/fileadmin/user_upload/images/CVD_Health/Global_CVD_Atlas.pdf. Accessed 15 Jan 2011.

[2] Mack M, Gopal A (2016). Epidemiology, traditional and novel risk factors in coronary artery disease. Heart Fail Clin.

[3] Brown MD (2003). Exercise and coronary vascular remodelling in the healthy heart. Exp Physiol.

[4] Bernardo BC, McMullen JR (2016). Molecular aspects of exercise-induced cardiac remodeling. Cardiol Clin.

[5] Tomanek RJ (1994). Exercise-induced coronary angiogenesis: a review. Med Sci Sports Exerc.

[6] Zhang J, Friedman MH (2013). Adaptive response of vascular endothelial cells to an acute increase in shear stress frequency. Am J Physiol Heart Circ Physiol.

[7] Smith SK (1998). Angiogenesis, vascular endothelial growth factor and the endometrium. Hum Reprod Update.

[8] Papanastasiou G, Williams MC, Dweck MR, Alam S, Cooper A, Mirsadraee S (2016). Quantitative assessment of myocardial blood flow in coronary artery disease by cardiovascular magnetic resonance: comparison of Fermi and distributed parameter modeling against invasive methods. J Cardiovasc Magn Reson.

[9] Marini M, Falcieri E, Margonato V, Treré D, Lapalombella R, di Tullio S (2008). Partial persistence of exercise-induced myocardial angiogenesis following 4-week detraining in the rat. Histochem Cell Biol.

[10] Kokoska L, Havlik J, Valterova I, Sovova H, Sajfrtova M, Jankovska I (2008). Comparison of chemical composition and antibacterial activity of *Nigella sativa* seed essential oils obtained by different extraction methods. J Food Prot.

[11] Cheikh-Rouhou S, Besbes S, Hentati B, Blecker C, Deoanne C, Attia H (2007). *Nigella sativa* L: chemical composition and physicochemical characteristics of lipid fraction. Food Chem.

[12] Hajhashemi V, Ghannadi A, Jafarabadi H (2004). Black cumin seed essential oil, as a potent analgesic and antiinflammatory drug. Phytother Res.

[13] Mansour MA, Nagi MN, El-Khatib AS, Al-Bekairi AM (2002). Effects of thymoquinone on antioxidant enzyme activities, lipid peroxidation and DT-diaphorase in different tissues of mice: a possible mechanism of action. Cell Biochem Funct.

[14] Hawsawi ZA, Ali BA, Bamosa AO (2001). Effect of *Nigella sativa* (black seed) and thymoquinone on blood glucose in albino rats. Ann Saudi Med.

[15] Ali BH, Blunden G (2003). Pharmacological and toxicological properties of *Nigella sativa*. Phytother Res.

[16] Dehkordi FR, Kamkhah AF (2008). Antihypertensive effect of *Nigella sativa* seed extract in patients with mild hypertension. Fundam Clin Pharmacol.

[17] Khattab MM, Nagi MN (2007). Thymoquinone supplementation attenuates hypertension and renal damage in nitric oxide deficient hypertensive rats. Phytother Res.

[18] El-Bahai MN, Al-Hariri MT, Yar T, Bamosa AO (2009). Cardiac inotropic and hypertrophic effects of *Nigella sativa* supplementation in rats. Int J Cardiol.

[19] Al-Asoom LI, Al-Shaikh BA, Bamosa AO, El-Bahai MN (2014). Effect of *Nigella sativa* supplementation to exercise training in a novel model of physiological cardiac hypertrophy. Cardiovasc Toxicol.

[20] Al-Asoom LI, Al-Shaikh BA, Bamosa AO, El-Bahai MN (2014). Comparison of *Nigella sativa*- and exercise-induced models of cardiac hypertrophy: structural and electrophysiological features. Cardiovasc Toxicol.

[21] Seif AA (2013). *Nigella sativa* Attenuates myocardial ischemic reperfusion injury in rats. J Physiol Biochem.

[22] Chen HI, Sharma B, Akerberg BN, Numi HJ, Kivelä R, Saharinen P (2014). The sinus venosus contributes to coronary vasculature through VEGFC-stimulated angiogenesis. Development.

[23] Al-Jassir M (1992). Chemical composition and microflora of black cumin (*Nigella sativa* L.) seeds growing in Saudi Arabia. Food Chem.

[24] Barbier J, Rannou-Bekono F, Marchais J, Tanguy S, Carré F (2007). Alterations of beta3-adrenoceptors expression and their myocardial functional effects in physiological model of chronic exercise-induced cardiac hypertrophy. Mol Cell Biochem.

[25] Lu J, Yao YY, Dai QM, Ma GS, Zhang SF, Cao L (2012). Erythropoietin attenuates cardiac dysfunction by increasing myocardial angiogenesis and inhibiting interstitial fibrosis in diabetic rats. Cardiovasc Diabetol.

[26] Ainscough JF, Drinkhill MJ, Sedo A, Turner NA, Brooke DA, Balmforth AJ (2009). Angiotensin II type-1 receptor activation in the adult heart causes blood pressure-independent hypertrophy and cardiac dysfunction. Cardiovasc Res.

[27] Zheng W, Seftor EA, Meininger CJ, Hendrix MJ, Tomanek RJ (2001). Mechanisms of coronary angiogenesis in response to stretch: role of VEGF and TGF-beta. Am J Physiol Heart Circ Physiol.

[28] Kolmakova A, Rajesh M, Zang D, Pili R, Chatterjee S (2009). VEGF recruits lactosylceramide to induce endothelial cell adhesion molecule expression and angiogenesis in vitro and in vivo. Glycoconj J.

[29] Peng YZ, Zheng K, Yang P, Wang Y, Li RJ, Li L (2015). Shock wave treatment enhances endothelial proliferation via autocrine vascular endothelial growth factor. Genet Mol Res.

[30] Nurro J, Halonen PJ, Kuivanen A, Tarkia M, Saraste A, Honkonen K (2016). AdVEGF-B186 and AdVEGF-DΔNΔC induce angiogenesis and increase perfusion in porcine myocardium. Heart.

[31] Randi AM, Laffan MA, Starke RD (2013). Von Willebrand factor, angiodysplasia and angiogenesis. Mediterr J Hematol Infect Dis.

[32] Starke RD, Ferraro F, Paschalaki KE, Dryden NH, McKinnon TA, Sutton RE (2011). Endothelial von Willebrand factor regulates angiogenesis. Blood.

[33] Bauditz J, Lochs H (2007). Angiogenesis and vascular malformations: antiangiogenic drugs for treatment of gastrointestinal bleeding. World J Gastroenterol.

[34] Cooke JP, Losordo DW (2002). Nitric oxide and angiogenesis. Circulation.

[35] Schirmer SH, Millenaar DN, Werner C, Schuh L, Degen A, Bettink SI (2015). Exercise promotes collateral artery growth mediated by monocytic nitric oxide. Arterioscler Thromb Vasc Biol.

[36] Kleindienst A, Battault S, Belaidi E, Tanguy S, Rosselin M, Boulghobra D (2016). Exercise does not activate the β3 adrenergic receptor-eNOS pathway, but reduces inducible NOS expression to protect the heart of obese diabetic mice. Basic Res Cardiol.

[37] Farah C, Kleindienst A, Bolea G, Meyer G, Gayrard S, Geny B (2013). Exercise-induced cardioprotection: a role for eNOS uncoupling and NO metabolites. Basic Res Cardiol.

[38] Feng D, Nagy JA, Pyne K, Dvorak HF, Dvorak AM (2004). Ultrastructural localization of platelet endothelial cell adhesion molecule (PECAM-1, CD31) in vascular endothelium. J Histochem Cytochem.

[39] Park S, Sorenson CM, Sheibani N (2015). PECAM-1 isoforms, eNOS and endoglin axis in regulation of angiogenesis. Clin Sci (Lond).

[40] DeLisser HM, Christofidou-Solomidou M, Strieter RM, Burdick MD, Robinson CS, Wexler RS (1997). Involvement of endothelial PECAM-1/CD31 in angiogenesis. Am J Pathol.

[41] Chodari L, Mohammadi M, Ghorbanzadeh V, Dariushnejad H, Mohaddes G (2016). Testosterone and voluntary exercise promote angiogenesis in hearts of rats with diabetes by enhancing expression of VEGF-A and SDF-1a. Can J Diabetes.

[42] Gielen S, Schuler G, Adams V (2010). Cardiovascular effects of exercise training: molecular mechanisms. Circulation.

